# Effects of direct current electric-field using ITO plate on breast cancer cell migration

**DOI:** 10.1186/2055-7124-18-10

**Published:** 2014-07-31

**Authors:** Min Sung Kim, Mi Hee Lee, Byeong-Ju Kwon, Hyok Jin Seo, Min-Ah Koo, Kyung Eun You, Dohyun Kim, Jong-Chul Park

**Affiliations:** Cellbiocontrol Laboratory, Department of Medical Engineering, Yonsei University College of Medicine, 134 Shinchon-dong, Seodaemun-gu, Seoul 120-752 Korea; Brain Korea 21 PLUS Project for Medical Science, Yonsei University College of Medicine, 134 Shinchon-dong, Seodaemun-gu, Seoul 120-752 Korea

**Keywords:** Cell migration, Electrotaxis, ITO plate, Breast cancer cell, MDA-MB-231

## Abstract

**Background:**

Cell migration is an essential activity of the cells in various biological phenomena. The evidence that electrotaxis plays important roles in many physiological phenomena is accumulating. In electrotaxis, cells move with a directional tendency toward the anode or cathode under direct-current electric fields. Indium tin oxide, commonly referred to as ITO has high luminous transmittance, high infrared reflectance, good electrical conductivity, excellent substrate adherence, hardness and chemical inertness and hence, have been widely and intensively studied for many years. Because of these properties of ITO films, the electrotaxis using ITO plate was evaluated.

**Results:**

Under the 0 V/cm condition, MDA-MB-231 migrated randomly in all directions. When 1 V/cm of dc EF was applied, cells moved toward anode. The y forward migration index was -0.046 ± 0.357 under the 0 V/cm and was 0.273 ± 0.231 under direct-current electric field of 1 V/cm. However, the migration speed of breast cancer cell was not affected by direct-current electric field using ITO plate.

**Conclusions:**

In this study, we designed a new electrotaxis system using an ITO coated glass and observed the migration of MDA-MB-231 on direct current electric-field of the ITO glass.

## Background

In a variety of biological phenomena, cell migration plays a very important role. Cellular migrations are prominent in morphogenic processes ranging from gastrulation to development of the nervous system in embryogenesis. Migration of fibroblasts and vascular endothelial cells is essential for wound healing. In metastasis, tumor cells immigrate from the initial tumor mass into the circulatory system, which they subsequently leave and migrate into a new site. In the inflammatory response, leukocytes migrate into the areas where insult has occurred, and then they affect phagocyte and immune functions. In normal physiology and pathology, migration remains crucial for the adult organism. Finally, cell migration is crucial to technological applications in tissue engineering and playing an essential role in colonization of biomaterials scaffolding [1, 2].

Most organs (especially glands) and embryos surrounded by a layer of epithelial cells produce potential differences or transepithelial potentials (TEPs) of a few millivolts to tens of millivolts. These correspond to transcellular direct-current EFs (dcEFs) of 50-500 mV/mm, as measured in vivo or in vitro in guinea pig trachea, mouse rectum, small airways of sheep lungs and rat prostate. Endogenous EFs might also exist in the central nervous system owing to the presence of extracellular field potentials across the blood–brain barrier, including specific transendothelial [3–9].

The evidence that electrotaxis is very important in many physiological phenomena is accumulating. The cells move with a directional preference toward the cathode or anode under direct-current electric fields (dcEFs) [10–14]. The preferential direction of migration during electrotaxis varies among the cell types and under different experimental conditions.

Indium tin oxide, commonly referred to as ITO, is an n-type semiconductor with a band gap between 3.5 and 4.3 eV and a maximum charge carrier concentration in the order of 10^21^ cm^−3^ [15–17]. Consequently, ITO is transparent to visible and near-infrared light and has a low electrical resistivity. ITO films have high luminous transmittance, high infrared reflectance, good electrical conductivity, excellent substrate adherence, hardness and chemical inertness and hence, have been widely and intensively studied for many years [17–20]. Because of these properties, ITO films are extensively used as coating electrodes in optoelectronic devices [21], electroluminescent devices [22], photovoltaic cells [23–25], electrochromic devices [21], liquid crystal displays [21–25], sensors [26], storage-type cathode ray tubes [21], biological devices [27], flat panel display devices and heat reflecting mirrors [28]. In this study, we designed a new electrotaxis system using an ITO glass where DC current flows on and observed the migration of MDA-MB-231 under this system.

## Methods

### Cell culture

MDA-MB-231 were purchased from ATCC (Rockville, MD, USA) and maintained in Dullbecco’s modification of eagle’s minimal essential medium (DMEM) supplemented with 10% fetal bovine serum (FBS, Lonza) and 1% anti-biotics. Cells were incubated at 37°C in 5% CO_2_ atmosphere and the medium was changed every 2-3 days. Cells were subcultured with 0.25% trypsin/EDTA when they reach 50 ~ 70% confluence.

### ITO plate electrotaxis system

**Figure 1:**
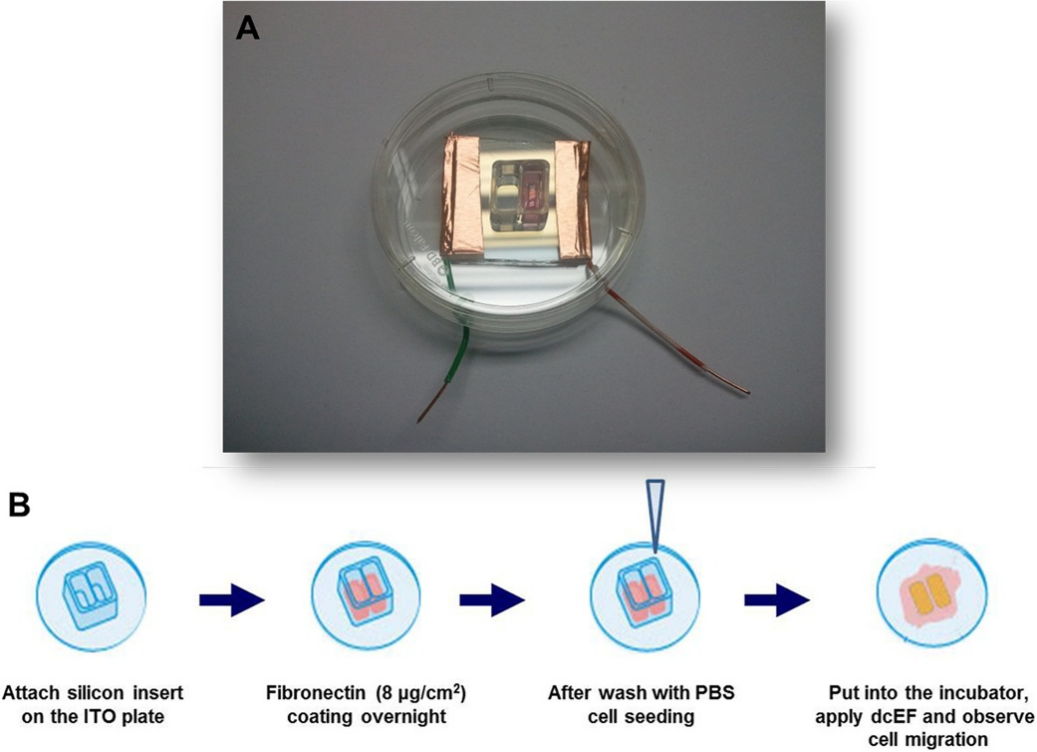
**Indium-tin-oxide (ITO) plate electrotaxis system.** Indium-tin-oxide (ITO) plate electrotaxis system **(A)** and Schematic diagram of electrotaxis using ITO plate **(B)**.

Electrotaxis system using Indium-Tin-Oxide coated glasses were designed for currents contact cells directly. Indium-Tin-oxide (ITO) coated glasses were kindly provided by Kwangwoon University and were cut with approximately dimensions 2 × 2 cm^2^. Cu tape (3 M, USA) connected both ends of 2 × 2 cm^2^ cut ITO coated glasses (ITO plate) with 5 cm long electric wire, then the ITO plate was dipped into 70% ethanol for 30 minutes. The sterilized grease were pasted to one side of the silicon culture insert (Ibidi, Munchen, Germany), then put the silicon insert on the ITO coated glass surface (Figure 1A).

### Electrotaxis of MDA-MB-231on ITO plate

Fibronectin needed to be coated on the surface of ITO plate for MDA-MB-231 cell attachment. Add 50 μl of fibronectin solutions (8 μg/cm^2^) to silicon insert and incubate overnight at 37°C. Cells (8 × 10^3^ cells/cm^2^) were seeded and allowed to grow for at least 24 hours in DMEM supplement with 10% FBS, 1% anti-biotics at 37°C in a 5% CO_2_ incubator (Figure 1B). Immediately before a test, medium was replaced with DMEM supplemented with 10% FBS, 1% anti-biotics. Cells were exposed to a direct-current electric field for 3 hours as indicated at 37°C in a temperature-controlled chamber on an inverted microscope stage.

### Cell viability assay

Cell viability was measured using LIVE/DEAD assay kit (Invitrogen, CA, USA). After electric field treatment, cells were washed 2 times with PBS, then they treated with resazurin 5 μM and SYTOX Green 0.1 μM in dark room. Cells were incubated in CO_2_ incubator for 15 minutes and The ITO plate were observed by a fluorescence inverted microscope.

### Time-lapse phase contrast microscopy and analysis of cell migration

**Figure 2:**
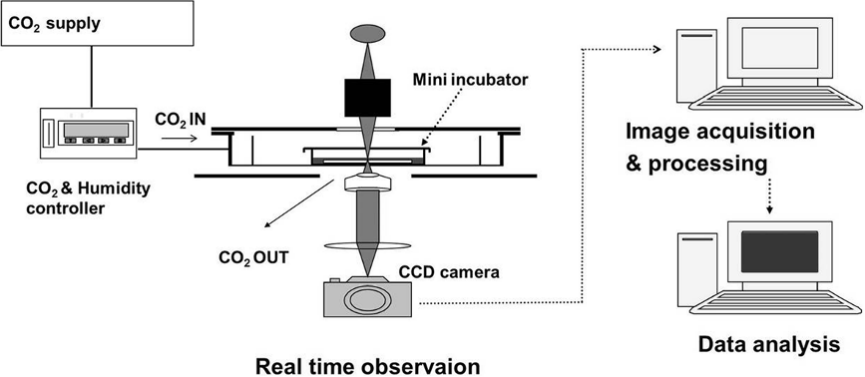
**Real time observation system.** Schematic diagram of the real time observation system for the evaluation of cell migration.

We used the real time observation system (Figure 2) to observe the migration of the cells on the ITO plate. The real time observation system consisted of incubator system installed with the microscope to observe live cells migration. The incubator was regulated by temperature and gas composition controlling program (CCP ver. 3.8, Live Cell Instrument, Korea) under proper environment for cell (CO_2_ 5%, 37°C).

### Image acquisition

The cells were cultured in the electrotaxis incubator placed on the microscope stage, and cell images were recorded every 5 minutes until electric treatment ends by the change-coupled device (CCD) camera (Electric Biomedical Co. Ltd., Osaka, Japan) attached to the inverted microscope (Olympus Optical Co. Ktd., Tokyo, Japan). Images were conveyed directly from a frame grabber to computer storage using Tomoro image capture program and saved them as JPEG image files.

### Cell tracking and evaluation of cell migration

For data analysis, captured images were imported into ImageJ (ImageJ 1.37v by W. Rusband, National Institutes of Health, Baltimore, Md). Image analysis was carried out by manual tracking and chemotaxis tool plug-in (v. 1.01, distributed by ibidi GmbH, Munchen, Germany) in ImageJ software. We obtained the datasets of XY coordinates by using manual tracking, then these datasets were imported into chemotaxis plug-in. This tool computed the cell migration speed and y forward migration index (y FMI) of cells and plotted the cell migration pathway. The migration speed was calculated as an accumulated distance of the cell divided by time. The y FMI of the cell was defined as the straight-line distance along the y axis between the start position and the end position of cell divided by accumulated distance. For each experiment, 20 cells were randomly selected along each edge of the wound. Cells undergoing division, death or migration outside the field of the view were excluded from the analysis.

## Results

### Viability of MDA-MB-231 on ITO plate

**Figure 3:**
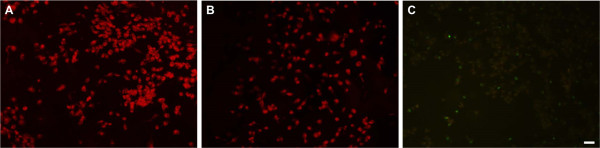
**LIVE/DEAD assay of MDA-MB-231.** LIVE/DEAD assay of MDA-MB-231 in an EF of 0 V/cm **(A)**, 1 V/cm **(B)** and 2 V/cm **(C)**. (Scale bar = 100 μm).

The appropriate strength of electric field needed to be determined before electrotaxis experiment on ITO plate. Figure 3 shows the viability of MDA-MB-231 on direct-current electric field of ITO glass. The cells under the electric field of 0 or 1 V/cm for 3 hours dyed red (Figure 3A and B). Most of cells under electric field of 2 V/cm showed little green fluorescence or slightly red fluorescence, some of them emitted bright green fluorescence (Figure 3C).

**Figure 4:**
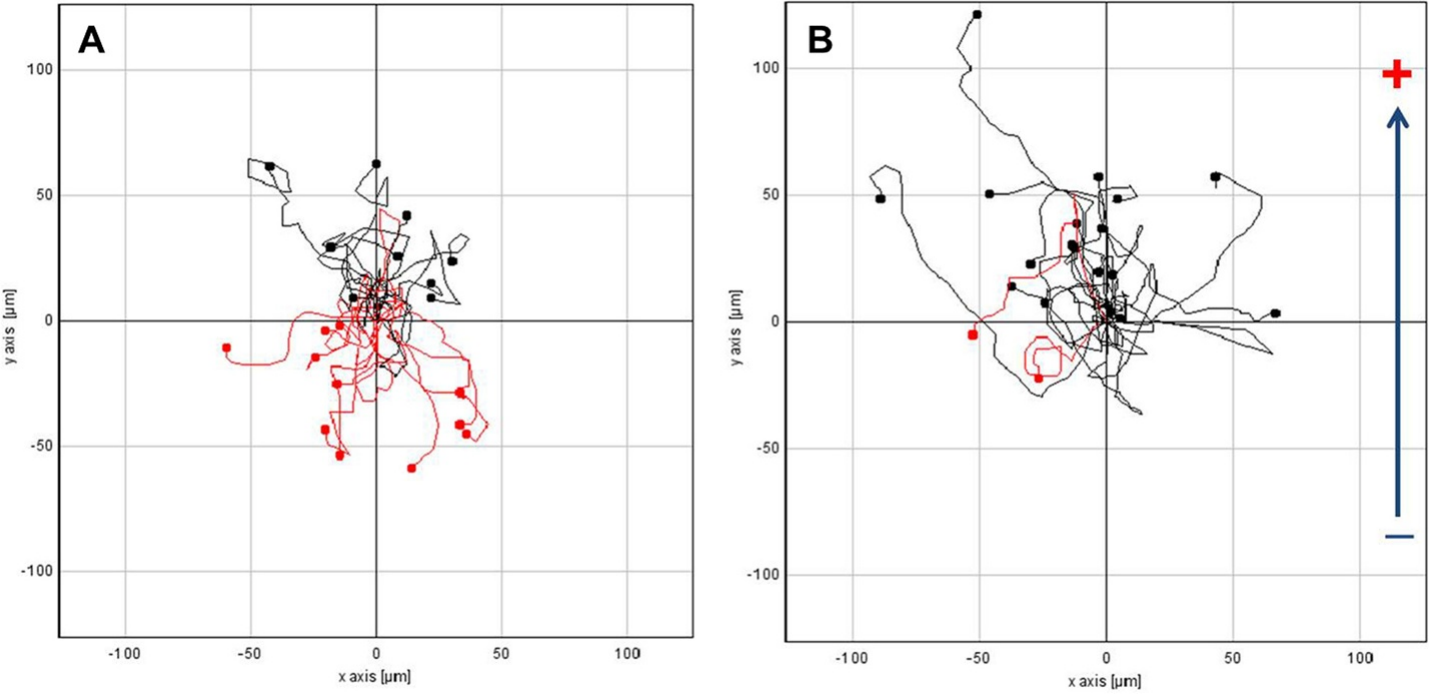
**Cell tracking image of MDA-MB-231.** Cell tracking image of MDA-MB-231 in an EF of 0 V/cm **(A)**, 1 V/cm **(B)**.

### Electrotaxis of MDA-MB-231 on ITO plate

**Figure 5:**
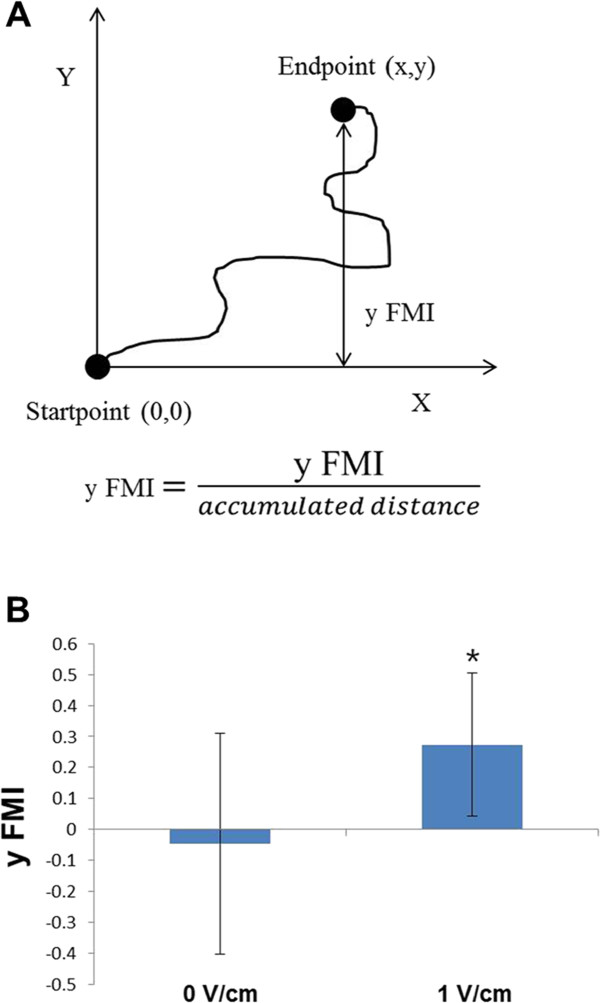
**The y forward migration index of MDA-MB-231.** Schematic diagram of the y FMI **(A)**, The y forward migration index of MDA-MB-231 in an EF of 0 V/cm and 1 V/cm **(B)** (*p < 0.05).

**Figure 6:**
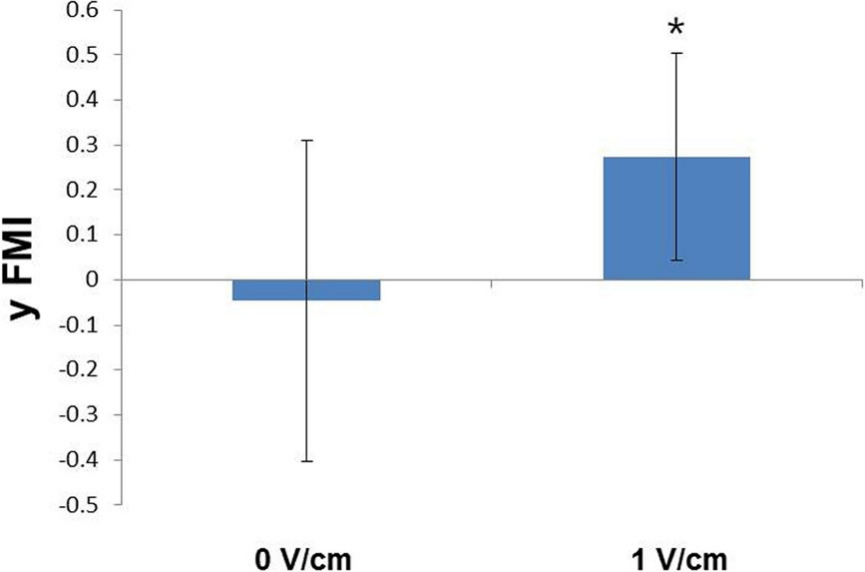
**The migration speed of MDA-MB-231.** The migration speed of MDA-MB-231 in an EF of 0 V/cm and 1 V/cm.

To make the electrotaxis system more convenient than the established, we considered Indium-tin-oxide coated slide glass. To identify the function of ITO glass electrotaxis system, MDA-MB-231 were seeded on the ITO plate and electrotaxis was evaluated. We confirmed the electrotaxis of MDA-MB-231 on ITO plate under direct current electric fields of 0 and 1 V/cm. In the stimulation free condition, cells migrated randomly in all directions with a scattered distribution (Figure 4A). When 1 V/cm of direct-current electric field was applied, MDA-MB-231 moved toward the anode (Figure 4B). To characterize the latency in directional cell migration, we analyzed the y forward migration index (y FMI) (Figure 5A). The y FMI value of 1 indicates the cell has migrated perfectly toward the anode, −1 means the cell has migrated perfectly toward the cathode and 0 indicates the cell has migrated perpendicular to the stimulation direction. The y FMI was -0.046 ± 0.357 under the 0 V/cm and was 0.273 ± 0.231 under dcEFs of 1 V/cm (Figure 5B). However, there were no statistical differences between the migration speed of cells under 0 V/cm (36.99 ± 9.40 μm/hr) and 1 V/cm (37.62 ± 16.23 μm/hr) (Figure 6).

## Discussion

The LIVE/DEAD assay kit provides two-color fluorescence assay that distinguishes metabolically active cells from injured cells and dead cells. The assay is based on the reduction of C12-resazurin to red-fluorescent C12-resorufin in metabolically active cells and the uptake of the cell-impermeant, green-fluorescent nucleic acid stain, SYTOX Green dye, in cells with compromised plasma membranes (usually late apoptotic and necrotic cells). The dead cells emit mostly green fluorescence whereas the healthy, metabolically active cells emit mostly red fluorescence. The injured cells have lower metabolic activity and, consequently, reduced red fluorescence emission; because they possess intact membranes, however, injured cells accumulate little SYTOX Green dye and, therefore, emit very little green fluorescence [29–31]. The more damaged cells were observed when the higher strength of direct-current electric field was applied. We chose 1 V/cm for the next experiment because most of cells were not damaged under 1 V/cm condition.

In human keratinocytes, the directionality was increased under the direct-current electric field while the migration speed was decreased [32]. This indicates that there is a difference between established electrotaxis system and ITO glass electrotaxis system, because only directionality was increased under direct-current electric field of ITO glass while migration speed was not changed. The established electrotaxis system used agar-salt bridges to applied the direct-current electric field through the media. In a new electrotaxis system using ITO glass, however, the cells contacting to ITO glass can directly be affected by direct-current electric field. To explain the directional migration of MDA-MB-231 on direct-current electric field of ITO glass and the differences between ITO glass system and existing electrotaxis system, further studies are required.

## Conclusions

In conclusion, MDA-MB-231 on the ITO film coated glass in direct-current electric fields migrated toward anode. Cell viability was dependent on the strength of direct-current electric field. The migration speed of MDA-MB-231 was not affected by the direct-current electric field using ITO plate. Therefore, the direct-current electric field using ITO glass induced the directional migration of breast cancer cell. Although further studies are required to figure out the differences between established electrotaxis and ITO glass electrotaxis system, it was identified that direct-current electric field of ITO glass affected the directional migration of breast cancer cell.

## Availability of supporting data

The data sets supporting the results of this article are included within the article.
